# Anaemia, hypoalbuminemia, and risk of cycloserine-associated neuropsychiatric toxicity in a shortened oral regimen for multidrug/rifampicin-resistant TB

**DOI:** 10.5588/ijtldopen.26.0098

**Published:** 2026-09-15

**Authors:** E.Q.L. Shen, M. Rashitov, E. Algozhina, N. Arlyapova, A. Skrahina, A. LaHood, L. Trevisi, E. Osso, M. Romo, K.J. Seung, C.D. Mitnick, M.L. Rich, M.F. Franke, Y. Algozhin

**Affiliations:** 1 Harvard Medical School, Boston, MA, USA;; 2 Partners In Health Kazakhstan, Almaty, Kazakhstan;; 3 Partners In Health, Boston, MA, USA;; 4 The Republican Scientific and Practical Centre for Pulmonology and Tuberculosis, Minsk, Belarus;; 5 Department of Global Health and Social Medicine, Harvard Medical School, Boston, MA, USA;; 6 Division of Global Health Equity, Department of Medicine, Brigham and Women’s Hospital, Boston, MA, USA;; 7 Department of Epidemiology, Harvard T.H. Chan School of Public Health, Boston, MA, USA.

**Keywords:** tuberculosis, MDR/RR-TB, drug toxicity, drug resistance, neuropsychiatric toxicity

## Abstract

**BACKGROUND:**

All-oral shortened regimens for multidrug/rifampicin-resistant TB (MDR/RR-TB) commonly include cycloserine, which is associated with neuropsychiatric toxicity. Predictors of cycloserine-related adverse events remain poorly defined. We assessed whether baseline anaemia or hypoalbuminemia predict neuropsychiatric toxicity.

**METHODS:**

We conducted a prospective cohort study of three 9-month standardised MDR/RR-TB regimens in Kazakhstan. Regimens shared a core of a fluoroquinolone, bedaquiline, and linezolid; one regimen included cycloserine. Neuropsychiatric adverse events of special interest (AESIs) included peripheral neuropathy, seizures, and psychosis. We conducted logistic regression to calculate associations between baseline anaemia and hypoalbuminemia and neuropsychiatric or central nervous system (CNS)-specific AESIs.

**RESULTS:**

In 556 cycloserine-treated individuals, 29 (5.2%) developed neuropsychiatric AESIs. Adjusted odds ratios comparing anaemia vs. no anaemia were 2.40 (95% confidence interval [CI]: 1.01–5.72) for neuropsychiatric AESIs and 2.71 (95% CI: 0.74–9.91) for CNS-specific AESIs. Adjusted odds ratios for grade ≥2 hypoalbuminemia were 3.59 (95% CI: 0.88–14.60) and 7.40 (95% CI: 1.58–34.66), respectively. In 146 individuals without cycloserine, neuropsychiatric AESI occurred in 0/49 with anaemia versus 5/97 without anaemia (0% vs 5.2%; *P* = 0.168), and in 1/3 with grade ≥2 hypoalbuminemia versus 3/90 without (33.3% vs 3.3%; Fisher’s exact *P* = 0.125).

**CONCLUSIONS:**

Baseline anaemia and hypoalbuminemia were associated with increased neuropsychiatric toxicity risk during treatment with a cycloserine-containing regimen. Whether these associations are specific to cycloserine merits further study.

All-oral shortened regimens have become an increasingly important strategy in the management of multidrug/rifampicin-resistant TB (MDR/RR-TB). Recent clinical trials and subsequent prospective cohort studies demonstrated strong efficacy and tolerability among several four- and five-drug regimens.^1–6^ These regimens share a common core of a fluoroquinolone, bedaquiline, and linezolid, supplemented by combinations of other drugs such as pretomanid, cycloserine–clofazimine (Cs-Cfz), delamanid–pyrazinamide (Dlm-Z), or delamanid–clofazimine (Dlm-Cfz).^1,3^ There is limited evidence to support decision-making around which combination is optimal for an individual patient. Cs is a second-tier agent commonly included in all-oral shortened treatment regimens and is notable for a unique neuropsychiatric profile. Associated adverse events include peripheral neuropathy, seizures, psychosis, and mood disturbances such as anxiety, depression, and suicidal ideation.^7,8^ Despite decades of clinical use, the exact neurotoxic mechanism of cycloserine remains uncertain.^8^ Proposed mechanisms include pyridoxine antagonism^9,10^ and partial agonist activity at central *N*-methyl-D-aspartate (NMDA) receptors.^11,12^ Pyridoxine supplementation, however, has not demonstrated clear systematic evidence for efficacy, and has actually been shown to be associated with increased peripheral neuropathy.^7^ Given the potential severity of cycloserine-associated toxicities, and the current lack of effective reversal or prophylactic interventions, it is important to identify factors which may predispose patients to an elevated risk of experiencing these events. As cycloserine is a narrow therapeutic index drug often being used in settings where routine therapeutic drug monitoring is not performed, identifying practical baseline predictors of cycloserine toxicity is also critical for improving risk-stratified monitoring strategies for those exposed to this drug.^13^

Haemoglobin and albumin measurements, serving as surrogates for several underlying physiologic vulnerabilities, including chronic inflammation and nutritional deficiency, may predict the risk of cycloserine toxicity. Inflammatory states and poor nutritional reserve have been associated with altered drug pharmacokinetics, increased blood–brain barrier permeability, and disrupted neurotransmitter signalling, including NMDA-mediated pathways which have been implicated in cycloserine neurotoxicity.^14–17^ Abnormalities in haemoglobin or albumin at treatment initiation may thus represent clinically accessible markers of physiologic frailty that predispose patients to cycloserine-associated neuropsychiatric events.

We conducted a prospective observational cohort study in Kazakhstan to assess the safety and effectiveness of three five-drug, all-oral regimens for MDR/RR-TB, one of which included cycloserine.^3^ Here, we analyse whether individuals with anaemia or hypoalbuminemia at the time of initiating a cycloserine-containing regimen experienced an increased risk of neuropsychiatric events.

## METHODS

Between 14 September 2020 and 29 June 2023, we enrolled patients ≥18 years old with bacteriologically confirmed MDR/RR pulmonary TB from five public health facilities in Kazakhstan as part of the STEM-TB multinational study (NCT05871489). A full list of exclusion criteria is found in the Supplementary Data. Given that the primary aims of STEM-TB were descriptive, the sample size was determined by the number of consenting individuals who initiated therapy for MDR/RR-TB at participating facilities during the study period. We evaluated three regimens with a shared core of bedaquiline–linezolid–levofloxacin (Bdq-Lzd-Lfx), each reinforced with two additional agents (Cs-Cfz, Dlm-Z, or Dlm-Cfz). Clinicians selected a regimen for individual participants with the intent of using only drugs that were ‘likely effective’, a designation based on confirmed TB susceptibility and each participant’s recent history of relevant anti-TB drug exposures. Patients with a prior history of neurologic or psychiatric conditions were not prescribed cycloserine at the start of treatment. Given that delamanid was not widely available, the Cs-Cfz regimen was used most frequently. Cycloserine was dosed by weight in accordance with the Order of the Ministry of Health of the Republic of Kazakhstan. Patients <45 kg received 500 mg daily, while those 45 kg or more received 750 mg daily.

### Study procedures

Upon enrolment, participants underwent a comprehensive medical assessment that included a medical history, clinical examination, pregnancy testing, sputum and blood collection, chest X-ray (CXR), and electrocardiogram (ECG). Laboratory evaluation included complete blood count and haemoglobin, creatinine/urea, electrolytes, albumin, liver function tests, and HIV/HBV/HCV screening. Participants completed monthly follow-up assessments throughout treatment, which consisted of a review of signs and symptoms, clinical examination, sputum smear microscopy/culture, haematology, biochemistry, and ECG. Pyridoxine supplementation was not prescribed routinely to patients taking cycloserine. If neuropsychiatric adverse events developed during treatment, cycloserine was discontinued and patients whose adverse reactions were attributed to this drug were administered pyridoxine supplementation.

### Adverse events reporting

Adverse events of special interest (AESIs) were pre-defined based on the known toxicities of agents in the deployed regimens, and included peripheral neuropathy, seizure, and psychosis. The severity thresholds, based on Médecins Sans Frontières (MSF) criteria, for which an adverse event was deemed clinically relevant are detailed in Supplementary Data Table S1.^18^ Peripheral neuropathy was assessed via the Brief Peripheral Neuropathy Screen. A diagnosis of neuropathy required a subjective symptom score >0, together with at least one abnormal bilateral objective finding (reduced vibratory sense or abnormal deep tendon ankle reflex).

### Exposure and outcome definitions

Baseline anaemia was defined according to WHO criteria (<13 g/dL for men >15 years of age, and <12 g/dL for women >15 years of age).^19^ Baseline hypoalbuminemia was classified according to the MSF grading scale (grade 1: <lower limit of normal to 3.0 g/dL, grade 2: <3.0–2.0 g/dL, grade 3: <2.0 g/L).^18^ We selected grade ≥2 hypoalbuminemia as the cut-off for subsequent analyses based on an emerging body of literature which has identified <3.0 g/dL as an inflection point for prediction of TB-related outcomes,^20–22^ and because this coincides with definitions for clinically significant levels of hypoalbuminemia when studied in other disease contexts.^23–25^ We defined neuropsychiatric AESIs to include clinically relevant episodes of peripheral neuropathy, seizures, and psychosis. We defined central nervous system (CNS)-AESIs as the specific subset of events including seizures and psychosis.

### Statistical analyses

We tabulated baseline characteristics in participants, stratified by whether they received a cycloserine-containing regimen. Next, we performed univariable logistic regression of baseline anaemia and hypoalbuminemia >grade 2 status on the development of a neuropsychiatric AESI or CNS-AESI among participants initiated on a cycloserine-containing regimen. We then conducted multivariable logistic regression analyses including both anaemia and hypoalbuminemia, and adjusting for age. Given that heavy alcohol use can cause anaemia and hypoalbuminemia and may exacerbate cycloserine-associated neurotoxicity by lowering the seizure threshold, we also included baseline alcohol use as a covariate to assess whether it could explain any observed association.^26^ We conducted sensitivity analyses using univariable and multivariable Cox proportional hazards regressions for time-to-development of a first neuropsychiatric AESI. To evaluate whether any observed associations were specific to cycloserine, we calculated associations among individuals who did not receive a cycloserine-containing regimen, serving as an internal negative comparator. Due to the small number of total neurotoxic events among those treated with a non-cycloserine regimen, we used contingency tables with Fisher’s exact testing to assess the distribution of neuropsychiatric or CNS-AESIs relative to anaemia and hypoalbuminemia status in this subgroup. All primary analyses were conducted in STATA 18 (College Station, TX: StataCorp LLC).

### Ethical statement

This study was approved by the Ethics Committee of the National Scientific Center for Phthisiopulmonology of the Ministry of Health of Kazakhstan and the Harvard Longwood Campus Institutional Review Board. All participants gave written informed consent.

## RESULTS

A total of 702 participants were enrolled. Among these, 556 (79.2%) participants initiated a cycloserine-containing regimen, of whom 29 (5.2%) developed neuropsychiatric AESIs during therapy: there were 17 cases of peripheral neuropathy, 7 cases of seizure, and 5 cases of psychosis. Of the 146 (20.8%) participants who initiated a non-cycloserine-containing regimen, 5 (3.4%) developed a neuropsychiatric AESI during treatment: there were four cases of peripheral neuropathy and one case of seizure (attributed to pyrazinamide). Participants in the Cs and non-Cs subgroups did not differ in age, sex, or prevalence of co-morbidities such as HIV, diabetes, substance use, and low body mass index (Table 1). No participants had baseline renal insufficiency (creatinine >1.5× upper limit of normal). Participants initiated on a non-cycloserine regimen were more likely to have baseline sputum smear positivity, sputum culture positivity, evidence of fibrosis on CXR, and previous use of first-line TB agents (Table 1).

**Table 1.** Baseline characteristics of individuals who initiated a shortened cycloserine-containing or non-cycloserine all-oral regimen for MDR/RR-TB, STEM-TB Kazakhstan cohort.

Characteristics	Overall (n = 702)	Regimen with CS (n = 556)	Regimen without CS (n = 146)
Demographics and clinical co-morbidities			
Age (median, IQR)	37 (28–50)	38 (28–50)	36 (28–49)
Female	285 (40.6)	221 (39.8)	64 (43.8)
Alcohol consumption (n = 698)	67 (9.6)	58 (10.5)	9 (6.3)
Actively smoking (n = 701)	145 (20.7)	120 (21.6)	25 (17.2)
HIV infection (n = 701)	16 (2.3)	13 (2.3)	3 (2.1)
CD4 count (median, IQR)	263 (110–369.5)	246 (89–372)	280 (200–291)
Diabetes	68 (9.7)	53 (9.5)	15 (10.3)
Low BMI (<18.5)	142 (20.2)	107 (19.2)	35 (24.0)
Hospitalised during initial treatment	572 (81.5)	452 (81.3)	120 (82.2)
Previous use of first-line agents	98 (14.0)	90 (16.2)	8 (5.5)
Baseline chest radiographic features			
Bilateral disease (n = 701)	175 (25.0)	137 (24.7)	38 (26.0)
Cavitary disease (n = 689)	337 (48.9)	269 (49.4)	68 (46.6)
Fibrosis (n = 682)	84 (12.3)	75 (13.9)	9 (6.3)
Baseline microbiologic features			
Sputum smear microscopy^A^ (n = 701)			
Negative	370 (52.8)	305 (55.0)	65 (44.5)
Scanty	67 (9.6)	45 (8.1)	22 (15.1)
1+	167 (23.8)	137 (24.6)	30 (20.6)
2+	51 (7.3)	36 (6.5)	15 (10.3)
3+	46 (6.6)	32 (5.8)	14 (9.6)
Culture positivity^A^ (n = 690)	470 (68.1)	359 (65.9)	111 (76.6)
Relevant baseline labs			
Baseline haemoglobin, g/dL (median, IQR)	13.0 (11.8–14.2)	13.0 (11.8–14.1)	13.1 (11.8–14.4)
Baseline anaemia^B^	243 (34.6)	194 (34.9)	49 (33.6)
Baseline albumin, g/L (median, IQR) (n = 578)	41.4 (24.2–46.0)	41 (38–46)	42.6 (38–45.9)
Grade 1 hypoalbuminemia^C^	39 (6.7)	33 (6.8)	6 (6.5)
Grade ≥2 hypoalbuminemia	19 (3.3)	16 (3.3)	3 (3.2)

CS = cycloserine; BMI = body mass index; IQR = interquartile range; MDR/RR-TB = multidrug/rifampicin-resistant TB; MSF = Médecins Sans Frontières.

A Based on sputum sample obtained within the window of −90 to +7 days from treatment initiation.

B Baseline anaemia defined according to WHO criteria (<13 g/dL for men >15 years of age and <12 g/dL for women >15 years of age).

C Hypoalbuminemia grades based on MSF scale (grade 1: <LLN to 3.0 g/dL, grade 2: <3.0–2.0 g/dL, grade 3: <2.0g/dL); LLN = lower limit of normal.

The distribution of baseline haemoglobin and albumin levels did not differ between those in the Cs and non-Cs subgroups (Table 1). Baseline anaemia was noted in 194 (34.9%) of participants initiated on a cycloserine-containing regimen, and 49 (33.6%) of participants initiated on a non-Cs regimen. A baseline albumin measurement was available for 578 (82.3%) participants. Of these, 16 (3.3%) participants in the Cs subgroup and 3 (3.2%) in the non-Cs subgroup had grade ≥2 baseline hypoalbuminemia. Missing baseline albumin values occurred due to transient lack of necessary laboratory reagents at certain clinical sites. Participants with and without baseline albumin did not differ in demographics or presence of baseline co-morbidities, though participants without an albumin measurement were less likely to have been hospitalised at baseline (Supplementary Data Table S2).

### Anaemia and AESIs in cycloserine-containing regimens

Participants with baseline anaemia had higher odds of developing a neuropsychiatric AESI (univariable odds ratio [OR]: 2.41, 95% confidence interval [CI]: 1.14–5.13) during treatment (Table 2). The magnitude of this association remained consistent in multivariable models that adjusted for age, self-reported alcohol use, and concurrent hypoalbuminemia (adjusted OR: 2.40, 95% CI: 1.01–5.72) – see Table 2. Similar results were observed with unadjusted and adjusted Cox proportional hazards regression of baseline anaemia status on time to first neuropsychiatric AESI or first CNS-specific AESI (Supplementary Data Table S3). Those with baseline anaemia had shorter times-to-first neuropsychiatric AESIs (adjusted hazard ratio [HR]: 2.32, 95% CI: 0.99–5.44) and CNS-AESIs (adjusted HR: 2.54, 95% CI: 0.70–9.18).

**Table 2.** Univariate and multivariable logistic regression of participant baseline anaemia and hypoalbuminemia status on development of a neuropsychiatric AESI during treatment with a shortened cycloserine-containing regimen for MDR/RR-TB, STEM-TB Kazakhstan cohort.

	Any neuropsychiatric AESI		CNS-specific AESI	
	Univariate logistic	Multivariable adjusted^A^ (n = 485)	Univariate logistic	Multivariable adjusted^A^ (n = 485)
Baseline anaemia^B^ (n = 555)	2.41 (1.14–5.13)	2.40 (1.01–5.72)	3.85 (1.14–12.95)	2.71 (0.74–9.91)
*P* value	0.022	0.048	0.029	0.131
Baseline hypoalbuminemia^C^ (n = 485)	4.93 (1.30–18.60)	3.59 (0.88–14.60)	11.79 (2.86–48.71)	7.40 (1.58–34.66)
*P* value	0.019	0.095	0.001	0.011

All cells displayed as logistic regression odds ratio (95% confidence interval).

AESI = adverse event of special interest; CNS = central nervous system; MDR/RR-TB = multidrug/rifampicin-resistant TB; MSF = Médecins Sans Frontières.

A Model with baseline anaemia and baseline hypoalbuminemia adjusted for each other, and additional covariates: age, self-reported alcohol use.

B Indicator for whether the participant had a haemoglobin result that met WHO criteria for anaemia, defined as <13 g/dL for men >15 years of age and <12 g/dL for women >15 years of age, within −60 to 30 days of the baseline visit date.

C Indicator for whether the participant had an albumin level which met MSF criteria for at least grade 2 hypoalbuminemia, within −60 to 30 days of the baseline visit date.

### Hypoalbuminemia and AESIs in cycloserine-containing regimens

Among participants with baseline hypoalbuminemia grade ≥2, the unadjusted and adjusted ORs for developing a neuropsychiatric AESI during treatment were 4.93 (95% CI: 1.30–18.60) and 3.59 (95% CI: 0.88–14.60), respectively, compared with those without (Table 2). When restricting to seizure and psychosis events, the adjusted OR was 7.40 (95% CI: 1.58–34.66) (Table 2), and in sensitivity analyses with Cox hazards regression, the adjusted OR was 6.48 (95% CI: 1.61–26.10) (Supplementary Table 3).

### Anaemia and hypoalbuminemia and AESIs in non-Cs containing regimens

In participants exposed to a non-cycloserine regimen (n = 146), we did not find evidence that individuals with anaemia were more likely to experience a neuropsychiatric AESI. No participants (of 49) with baseline anaemia, and 5 of 97 participants (5.2%) without anaemia, developed a neuropsychiatric AESI (Fisher’s exact *P* = 0.168). Neuropsychiatric AESIs occurred in a greater proportion of participants with grade ≥2 hypoalbuminemia (1 of 3, 33.3%), in comparison to those without (3 of 90, 3.3%) (Fisher’s exact *P* = 0.125).

## DISCUSSION

Neuropsychiatric adverse events were uncommon overall across three all-oral shortened regimens deployed for pulmonary MDR/RR-TB in Kazakhstan, and lower than rates reported by several previous studies of longer MDR-TB regimens (9%–11%).^27–29^ However, among patients treated with cycloserine, those with anaemia or hypoalbuminemia experienced a higher risk of neurotoxicity and CNS-specific neurotoxicity.

We did not find a positive association between anaemia and neurotoxicity in regimens that excluded cycloserine, supporting our hypothesis that the relationship may be cycloserine-specific. However, because fewer than 150 individuals received regimens without cycloserine, these findings should be interpreted with caution. Hypoalbuminemia, by contrast, was associated with a higher proportion of neuropsychiatric events in both cycloserine and non-cycloserine regimens, though interpretation in the latter group was limited by the small number of events. This pattern suggests that albumin status may portend a generally increased risk for drug-related neurotoxicity, and not be specific to cycloserine. Linezolid was used in all regimens, and is a recognised cause of peripheral neuropathy. Given that hypoalbuminemia has been associated with linezolid overexposure, potentially via increased free linezolid concentrations, it is possible that the association between hypoalbuminemia and neurotoxic events was driven by increased risk for supratherapeutic linezolid levels.^30–33^

These findings offer insight into two areas. First, they provide empirical evidence of physiologic vulnerabilities, captured here by anaemia and hypoalbuminemia, as markers of increased neurotoxicity risk. One explanation is that patients with chronic inflammation or malnutrition, both of which can drive anaemia and hypoalbuminemia, may also experience changes in CNS metabolism, neurotransmitter signalling, or blood–brain-barrier integrity.^15,16^ These changes may in turn potentiate the neurotoxic effects of cycloserine, whether through altered CNS drug concentrations or decreased neural resilience, particularly in the context of the drug’s narrow therapeutic index. Anaemia and hypoalbuminemia are easily evaluated and may serve as indicators of underlying drivers such as micronutrient deficiencies or heavy alcohol use, which may themselves be unmeasured or measured imperfectly. Further studies are needed to clarify the mechanisms underlying these correlations. Second, our results offer preliminary evidence that may support clinical decision-making. As efficacy across multiple four- and five-drug regimens for pulmonary MDR/RR-TB appears comparable based on existing clinical trial and observational data, safety and tolerability may serve as important differentiators in regimen selection.^1,3^ Given the lack of clear systematic evidence demonstrating pyridoxine supplementation as an effective means for reducing cycloserine-related events, it is important to consider strategies for identifying patients at high-risk and limiting their exposure. In this light, the presence of severe baseline anaemia may warrant preferential use of a non-cycloserine-containing regimen when alternative options are available. This approach may be particularly valuable in settings where close neurologic monitoring is challenging, therapeutic drug monitoring is not performed, or where underlying nutritional or chronic inflammatory co-morbidities are common.^34,35^ Given the common use of linezolid across these shortened regimens and established concerns of linezolid-associated adverse events in patients with known anaemia, particular attention may be warranted for individuals with baseline anaemia who are exposed to both linezolid and cycloserine during TB therapy.

Our study is limited by a fortunately low number of total neuropsychiatric adverse events, which led to wide CIs and reduced power for some analyses, particularly within the smaller non-cycloserine arm. These small numbers precluded formal statistical testing for effect modification by receipt of a cycloserine-containing regimen. Due to lack of drug monitoring, we are also unable to identify whether anaemia and hypoalbuminemia were associated with increased toxicity due to supratherapeutic cycloserine concentrations, or markers of clinical susceptibility despite normotherapeutic drug levels.

The observed results provide early evidence of risk factors for neuropsychiatric toxicity that are detectable on routine baseline laboratory evaluation; additional analysis in larger studies is needed to determine whether our findings are specific to cycloserine-containing regimens, and whether there may be any similar implications for terizidone-containing regimens. To reduce neuropsychiatric risk during MDR/RR-TB treatment, it is possible that clinicians could consider using non-Cs regimens in patients with notable baseline haematologic abnormalities. Targeted cycloserine drug monitoring for patients with anaemia and/or hypoalbuminemia may also be considered as a future strategy to reduce preventable toxicity while maintaining regimen effectiveness. Further research is needed to delineate the pathophysiology of cycloserine neurotoxicity and to validate these noted associations across other patient cohorts.

## Supplementary Material
