# Supplementary material for: Anaemia, hypoalbuminemia, and risk of cycloserine-associated neuropsychiatric toxicity in a shortened oral regimen for multidrug/rifampicin-resistant TB
Source: IJTLD Open. 2026 Sep 15;3(9):592–7. doi: 10.5588/ijtldopen.26.0098 (PMC13577210; doi:10.5588/ijtldopen.26.0098)
Supplement: Supplementary file 1 [file ijtldopen26-0098_supplementarydata1.pdf]

<u>SUPPLEMENTARY MATERIAL</u>

Anemia, Hypoalbuminemia and Risk of Cycloserine-Associated Toxicity in a Shortened Oral Regimen for MDR/RR-TB

Supplementary Table 1. Adverse Events of Special Interest and Severity Scale Threshold for Clinically Relevant Adverse Events of Special Interest, STEM-TB Kazakhstan Cohort

AESI	Severity Scale or “Other” Terms Included	Grade(s)	Definition for Minimum Grade to Be Considered Clinically Relevant
Hepatotoxicity	Alanine aminotransferase (ALT or SGPT) increased	≥ 3	Grade 3: alanine aminotransferase and/or aspartate aminotransferase >5 times the upper limit of normal
	Aspartate aminotransferase (AST or SGOT) increased	..	..
	Hepatotoxicity	..	..
	Hepatitis	..	..
Myelosuppression	Anemia	Anemia ≥ 3	Grade 3: hemoglobin < 7.9 g/dL
	Platelets decreased	Thrombocytopenia ≥ 3	Grade 3: platelets decreased <50,000/mm^3
	White blood cells decreased	Leukopenia ≥ 3	Grade 3: white blood cells decreased <2000/mm^3
	Lymphocyte count decreased	Lymphocytopenia ≥ 3	Grade 3: lymphocytes decreased <500/mm^3
	Absolute neutrophil count	Neutropenia ≥ 2	Grade 2: absolute neutrophile count <750/mm^3
	Pancytopenia	Pancytopenia ≥ 2	Grade 2: any combination of the above
Optic neuritis	Optic nerve disorder (optic neuritis)	All grades	Grade 1: clinical diagnosis, no symptoms
Peripheral neuropathy	Neurosensory disorders	All grades	Grade 1: mild impairment or discomfort / Brief Peripheral Neuropathy Screen subjective sensory neuropathy score 1-3 on any side
	Paresthesia (burning, tingling, etc.)	..	..
QT prolongation	Electrocardiogram QT-corrected interval prolonged	≥ 3	Grade 3: QTc≥501 msec, no symptoms
Seizures	Seizure	All grades	Grade 1: brief partial seizure, no loss of consciousness

Abbreviations: AESI, adverse event of special interest; QTc, QT interval corrected by the Fridericia method; SGOT, serum glutamic oxaloacetic transaminase; SGPT, serum glutamate pyruvate transaminase; ALT, alanine aminotransferase; AST, aspartate amin

Supplementary Table 2. Baseline Characteristics of Individuals Who Initiated a Shortened All-Oral Regimen for MDR/RR-TB, Stratified by Whether Participant Had a Baseline Albumin Measurement, STEM-TB Kazakhstan Cohort

Characteristics	Not Missing baseline albumin (n = 578)	Missing baseline albumin (n = 124)
Demographics and Clinical Comorbidities		
Age (median, IQR)	37 (28-50)	37.5 (26.5-51)
Female	234 (40.5)	51 (41.1)
Alcohol consumption (n = 698)	62 (10.7)	5 (4.1)
Actively smoking (n = 701)	119 (20.6)	26 (21.0)
HIV infection (n = 701)	15 (2.6)	1 (0.8)
CD4 count (median, IQR)	280 (89-372)	200
Diabetes	56 (9.7)	12 (9.7)
Low BMI (<18.5)	117 (20.2)	25 (20.2)
Hospitalized during initial treatment	493 (85.3)	79 (63.7)
Previous use of first-line agents	84 (14.5)	14 (11.3)
Baseline Chest Radiographic Features		
Bilateral disease (n = 701)	148 (25.6)	27 (21.9)
Cavitary disease (n = 689)	285 (50.3)	52 (42.6)
Fibrosis (n = 682)	83 (14.7)	1 (0.8)
Baseline Microbiologic Features		
Sputum Smear Microscopy[1] (n = 701)		
Negative	297 (51.5)	73 (58.9)
Scanty	62 (10.7)	5 (4.0)
1+	144 (25.0)	23 (18.6)
2+	41 (7.1)	10 (8.1)
3+	33 (5.7)	13 (10.5)
Sputum Culture Positivity[1] (n = 690)	391 (68.7)	79 (65.3)
Relevant Baseline Labs		
Baseline Hemoglobin, g/dL (median, IQR)	13.0 (11.8-14.1)	13.1 (11.8-14.3)
*Baseline Anemia**	202 (34.9)	41 (33.1)

* Baseline anemia defined according to WHO criteria (<13 g/dL for males >15 years of age and <12 g/dL for females >15 years of age)

[1] Based on sputum sample obtained within the window of -90 days to +7 days from treatment initiation

Supplementary Table 3. Univariate and Multivariable Cox Proportional Hazards Regression of Participant Baseline Anemia and Hypoalbuminemia Status on Development of a Neuropsychiatric AESI During Treatment with a Cs-Containing Shortened All-Oral Regimen for MDR/RR-TB, STEM-TB Kazakhstan Cohort

	Any Neuropsychiatric AESI		CNS-Specific AESI	
	Unadjusted HR	Adjusted[1] HR	Unadjusted HR	Adjusted[1] HR
Baseline anemia[+] (n = 556)	2.37 (1.14 - 4.92)	2.32 (0.99 - 5.44)	3.78 (1.14 - 12.56)	2.54 (0.70 - 9.18)
P value	*0.021*	*0.053*	*0.03*	*0.155*
Baseline hypoalbuminemia[++] (n = 485)	4.54 (1.35 - 15.22)	2.89 (0.80 - 10.47)	10.27 (2.78 - 37.94)	6.48 (1.61 - 26.10)
P value	*0.014*	*0.106*	*<0.001*	*0.009*

[+] Indicator for whether the participant had a hemoglobin result that met WHO criteria for anemia, defined as <13 g/dL for males >15 years of age and <12 g/dL for females >15 years of age, within -60 to 30 days of the baseline visit date

[++] Indicator for whether the participant had an albumin level which met MSF criteria for at least grade 2 hypoalbuminemia, within -60 to 30 days of the baseline visit date

[1] Model with baseline anemia and baseline hypoalbuminemia adjusted for each other, and additional covariates: age, self-reported alcohol use at baseline

Appendix A. Exclusion Criteria Against Initiating a Shortened All-Oral Regimen for MDR/RR-TB, STEM-TB Kazakhstan Cohort

- Younger than 18 years of age
- Unable to take oral medication
- Taking any medications contraindicated with drugs in the 9-month regimens
- Known allergy or hypersensitivity to a drug in the 9-month regimens
- Any prior documented resistance to a fluoroquinolone (or other drugs for which there was a reliable drug susceptibility test)
- Previous treatment with 1 or more second-line drugs in the regimen (>1 month)
- Extrapulmonary TB without pulmonary TB
- Severe or intractable extrapulmonary TB (e.g. TB meningitis or miliary TB)
- Baseline Fridericia-corrected QT interval ≥500ms on electrocardiogram, despite correction of serum electrolytes
- Baseline aspartate aminotransferase or alanine aminotransferase greater than 3 times the upper limit of normal
- Baseline creatinine clearance below 30 mL/minute per 1.73 m^2 body surface area
